# Stimuli-responsive nanozyme-hydrogel systems for ischemic stroke: toward spatiotemporal catalytic control of the neurovascular unit

**DOI:** 10.3389/fneur.2026.1945621

**Published:** 2026-08-26

**Authors:** Sen Yan, Xiaohong Wang, Cai Li, Rong Wang

**Affiliations:** 1 Qilu Medical University, Zibo, China; 2 Hebei Normal University, Shijiazhuang, China

**Keywords:** blood-brain barrier, catalytic therapy, ischemic stroke, nanozyme, neurovascular unit, reperfusion injury, smart biomaterials, stimuli-responsive hydrogel

## Abstract

The development of ischemic stroke involves swiftly changing and spatially varied states, including oxidative burst, acidosis, hypoxia, endothelial dysfunction, blood-brain barrier disruption, neuroinflammation, and later tissue remodeling. This temporal structure is poorly matched by conventional single-dose neuroprotectants. Nanozyme-hydrogel systems that respond to stimuli provide a promising approach by integrating catalytic redox control with localized retention, lesion-adaptive mechanics, and release triggered by specific cues. This Mini Review critically examines the design logic of such systems for ischemic stroke. Initially, we categorize reactive oxygen and nitrogen species, low pH, hypoxia, thrombin, matrix metalloproteinases, and inflammatory signals as a pathological code for activating materials. Following this, we examine nanozyme catalytic modules, responsive hydrogel matrices, and integration approaches including encapsulation, anchoring, *in situ* assembly, and multi-input gating. Emphasis is on the phase-aligned management of the neurovascular unit, covering early reperfusion defense, endothelial stabilization, glial adjustment, angiogenesis, and neural reconfiguration. A key takeaway is that there is limited direct evidence of fully integrated nanozyme-hydrogel platforms specifically for stroke; the field largely relies on the intersection of advanced stroke nanozymes and responsive hydrogels. Consequently, we recommend translational benchmarks that emphasize phase-route alignment, exact catalytic activity, degradation tracking, production feasibility, and validation in various preclinical settings. Accordingly, this Mini Review is intended as a design framework for hypothesis generation and preclinical validation, rather than as evidence of clinical readiness.

## 1 Introduction

Reperfusion continues to be the primary approach in treating acute ischemic stroke, but even successful recanalization cannot prevent secondary injury or guarantee tissue recovery (1). The reintroduction of oxygen into tissue with metabolic issues can worsen mitochondrial dysfunction, increase the production of reactive oxygen and nitrogen species (ROS/RNS), cause endothelial damage, lead to blood-brain barrier (BBB) breakdown, attract leukocytes, and result in swelling (2). At the same time, the lesion is chemically heterogeneous: acidosis develops with bioenergetic failure (3), hypoxia-responsive pathways change with injury severity and duration (4), and the focus within the cells gradually moves from neurones in immediate danger to a malfunctioning neurovascular unit (NVU) that includes endothelial cells, pericytes, astrocytes, microglia, neurones and the extracellular matrix (5).

This biology creates a strong rationale for materials that respond to lesion state rather than release a fixed dose on a fixed schedule. Stroke nanomedicine reviews have recently documented systems that are reactive to ROS, pH, hypoxia, thrombin, and matrix metalloproteinases (MMPs) (6, 7). In parallel, for neurological disorders, nanozymes have emerged as long-lasting enzyme-like catalysts, with activities such as superoxide dismutase, catalase, and peroxidase that can be fine-tuned by their composition and structure (8, 9). Hydrogels have the additional advantage of local retention of therapeutic components, their ability to conform to irregular brain cavities, control diffusion and provide a permissive matrix for repair.

**Figure 1:**
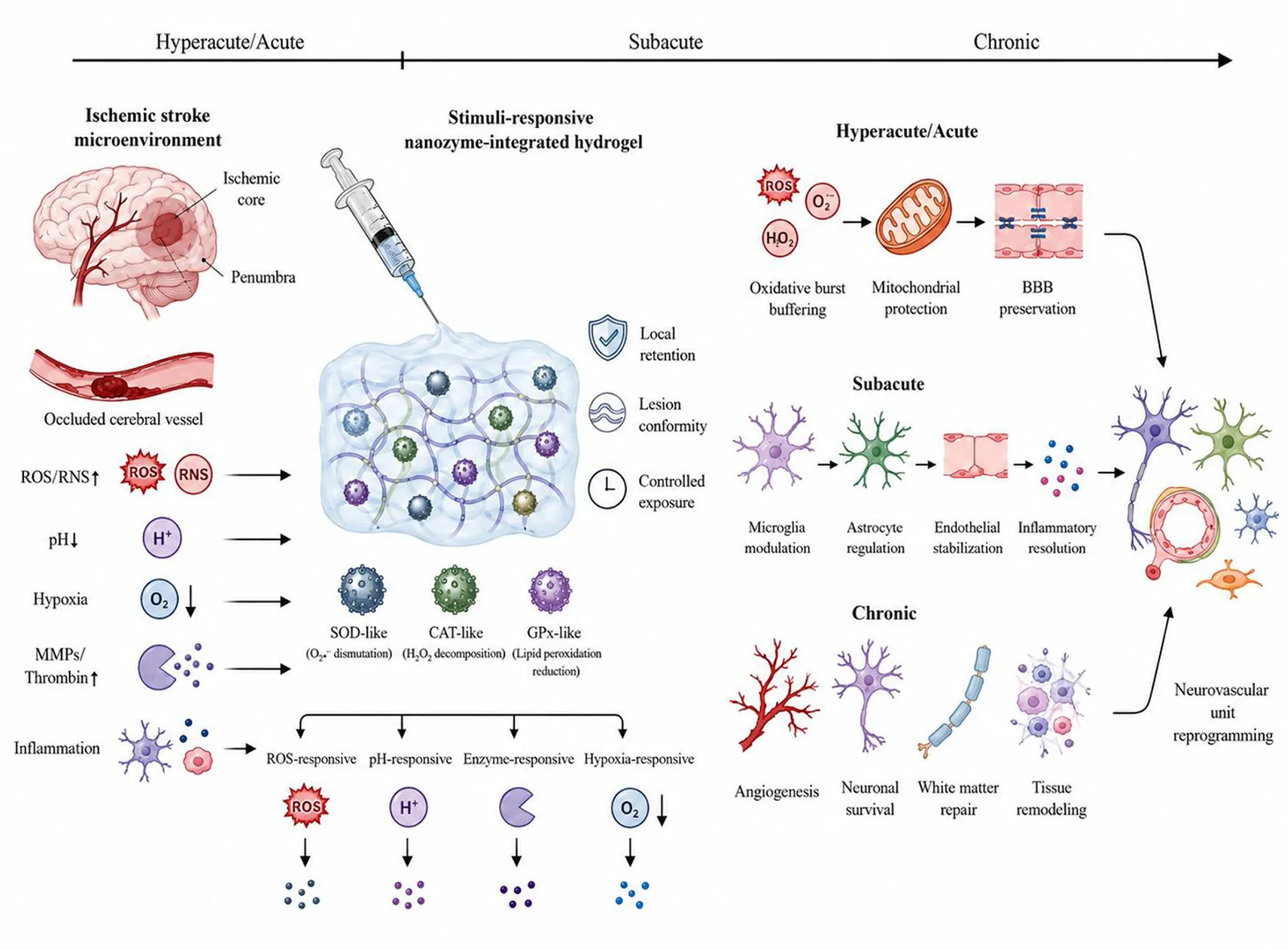
Spatiotemporal therapeutic logic of stimuli-responsive nanozyme-integrated hydrogels for ischemic stroke.

The combination appears intuitively compelling, but the evidence base must be described accurately. Stroke-specific nanozyme research is largely based on nanoparticle systems, in contrast to stroke hydrogel studies, which mainly deliver drugs, gases, proteins, cells, or extracellular vesicles. Integrated nanozyme-hydrogel systems have been proven effective in other central nervous system injuries, yet they are not commonly used for ischemic stroke. In this Mini Review, nanozyme-hydrogel therapy is viewed as an emerging convergence, not yet a defined clinical class. The discussion centers on the rational contributions of both modules, the mechanistic justification for their integration, and the evidence available. The resulting phase-matched design framework is summarized in Figure 1. The review therefore distinguishes stroke-specific evidence from mechanistic support extrapolated from other central nervous system injuries.

In an ischemic stroke, the surrounding environment is defined by an abundance of reactive oxygen and nitrogen species (ROS/RNS), acidic conditions, low oxygen levels, increased matrix metalloproteinase (MMP) and thrombin activity, and amplified inflammation. These endogenous pathological cues can be exploited to activate stimuli-responsive nanozyme-integrated hydrogels. Providing local retention, lesion conformity, and regulated exposure, the hydrogel matrix contains nanozymes that perform catalytic activities similar to superoxide dismutase (SOD), catalase (CAT), and glutathione peroxidase (GPx). ROS-, pH-, enzyme-, and hypoxia-responsive mechanisms further enable lesion-adaptive activation and release. In the hyperacute and acute stages, the main therapeutic focus is on quickly neutralizing the oxidative burst, safeguarding mitochondria, and maintaining the integrity of the blood-brain barrier (BBB). During the subacute phase, sustained microenvironmental regulation supports microglial modulation, astrocyte regulation, endothelial stabilization, and inflammatory resolution. In the chronic phase, treatment efforts concentrate on promoting angiogenesis, ensuring neuronal survival, repairing white matter, and remodeling tissue. Collectively, these phase-matched effects converge on reprogramming of the neurovascular unit and restoration of multicellular neurovascular homeostasis.

## 2 The ischemic microenvironment as a design code

An effective endogenous trigger doesn't have to be the most atypical signal; rather, it should be enriched, spatially informative, and synchronized with therapeutic aims. ROS/RNS are attractive because reperfusion can generate a high oxidative burden across neurons, mitochondria, endothelium, and the BBB (2). However, ROS also function as physiological signaling molecules. Consequently, an antioxidant material that is constantly active might inhibit reparative redox signaling following the acute phase. The design goal should focus on limited catalytic buffering instead of maximum scavenging.

Acidosis serves as another factor. Ischemic tissue can become significantly acidic, whereas the areas around the infarct exhibit milder and changing pH levels (3). The release in injured tissue can be hastened by acid-labile bonds, swelling that relies on protonation, or catalytic activity that shifts with pH, though pH by itself is unlikely to accurately indicate the stage of a lesion. Hypoxia-responsive chemistry is similarly attractive but complicated by the context-dependent roles of hypoxia-inducible factor signaling (4). Enzymatic signals might provide more precise mechanisms: thrombin is directly associated with thrombosis and vascular damage, while MMP activity indicates matrix remodeling and BBB disruption. Inflammatory mediators and cell trafficking can provide additional biological gates, but they are heterogeneous and may change from damaging to reparative states.

These factors support the use of designs with multiple inputs. A material that responds to both ROS and acidity, or links thrombin detection to a following catalytic action, is theoretically more selective than a system with only one trigger (6, 7). The importance of spatial heterogeneity is on par. The ischemic core, salvageable penumbra, perivascular compartments, and microvessels that are not fully reperfused differ in substrate concentrations and therapeutic priorities. *In vivo*, a response threshold adjusted in a homogeneous buffer might activate too early in mildly stressed tissue or too late in a rapidly deteriorating core. For this reason, trigger thresholds, diffusion distances, and catalytic substrate access should be mapped against tissue compartments rather than reported only as bulk responsiveness.

Over time, the correct response also evolves. During the hyperacute and early acute stages of injury, the emphasis is on swiftly controlling oxidative amplification and vascular failure. In the subacute phase, the focus shifts to immune-state changes, BBB restoration, and matrix remodeling. Subsequently, angiogenesis, neural preservation, white-matter restoration, and circuit plasticity take precedence. Table 1 summarizes a phase-matched design logic for future nanozyme-hydrogel systems.

**Table 1:** Phase-matched design logi c for stimuli-responsive nanozyme–hydrogel systems in ischemic stroke.

Phase/cue	Material logic	Intended NVU output	Critical caveat
Hyperacute/reperfusion; ROS/RNS surge ± acidity (2, 3)	Fast catalytic buffering; ROS- or pH-responsive exposure (6, 7, 11–18)	Preserve endothelium, BBB integrity, and viable neurons (15, 24, 25)	Must activate within minutes–hours; avoid over-scavenging of physiological redox signals (2, 3, 6, 7)
Acute thrombotic/vascular; thrombin ± oxidative stress (10)	Thrombin-triggered targeting or activation coupled to catalysis (10)	Thrombus control plus vascular and reperfusion protection (10, 30)	Must remain compatible with thrombolysis or thrombectomy and avoid added bleeding risk (10)
Acute–subacute matrix injury; MMP activity (19)	MMP-cleavable matrix or controlled release (19)	Barrier repair and later angiogenic support (19, 24–26)	MMP heterogeneity may cause premature degradation or off-target release (19)
Subacute inflammation; ROS plus inflammatory state (27, 28)	Sustained local catalysis followed by reduced intensity (15, 17, 18, 20)	Recalibrate microglia–astrocyte signaling and limit secondary injury (20, 27, 28)	Avoid indiscriminate immunosuppression and preserve reparative responses (27, 28)
Repair/remodeling; resolving oxidative stress plus tissue cavity (16, 21, 22, 29)	Porous or sequential hydrogel; low catalytic intensity; regenerative cue exposure (16, 21–23, 29)	Angiogenesis, neural progenitor integration, and tissue reconstruction (21, 22, 29)	Chronic persistence, foreign-body response, and material fate must be defined (21, 22, 31, 32)

BBB, blood–brain barrier; MMP, matrix metalloproteinase; NVU, neurovascular unit; ROS/RNS, reactive oxygen and nitrogen species.

## 3 Engineering nanozyme-hydrogel systems

### 3.1 Nanozymes as catalytic modules

The primary benefit of a nanozyme is its ability to catalyze reactions repeatedly. In contrast to stoichiometric antioxidants that are used up during the neutralization process, a properly designed nanozyme can continuously convert reactive substrates. This is relevant to reperfusion injury where multiple ROS/RNS species are continuously produced and conventional antioxidants suffer from short half-lives, poor brain access and limited target retention (8, 9).

Research on strokes shows a swift shift from general scavenging to targeted catalysis in lesions. A nanozyme templated by a thrombin-activated peptide links thrombus-specific activation with thrombolytic and neuroprotective benefits (10). Dual-Fe-atom nanozymes coated with Se-containing metal-organic frameworks were designed for multi-enzyme cascade activity in cerebral ischemia-reperfusion injury (11). Transferrin-mediated manganese-based nanozymes addressed BBB transport and ROS rebalancing (12), and Co-doped Fe3O4 nanozymes scavenged several ROS/RNS species (13). The idea was then extended to organelle level protection with mitochondria-targeted ceria nanoenzymes (14). Recently, a biomimetic nanozyme aimed at the brain was found to protect the NVU, associating catalytic treatment with the preservation of the BBB and the moderation of inflammation, rather than just addressing a single oxidative endpoint (15).

The examples highlight three design guidelines. The first is that catalytic activity should be evaluated in biologically relevant environments, since protein adsorption, pH, ionic strength, and native substrates can modify the observed enzyme-mimetic activity. Second, the presence of multiple enzyme activities does not guarantee superiority; catalytic promiscuity can produce unwanted intermediates. Third, targeting and activation should be seen as essential components of catalysis, rather than supplementary features. A highly active catalyst that is diluted systemically or retained in off-target organs is not a precise stroke therapy.

### 3.2 Responsive hydrogels as spatial and temporal modules

The challenge hydrogels tackle is the location and duration of therapeutic function expression. A conductive injectable hydrogel, resembling the extracellular matrix, was developed for sequential stroke treatment (16). In response to the ischemic microenvironment, a hydrogel with two crosslinks provided rapid anti-inflammatory effects alongside extended nitric oxide delivery for vascular and neural recovery (17). Various multifunctional injectable hydrogels have been utilized to alter the pathological microenvironment following ischemia (18). MMP-responsive nanofiber hydrogels exhibit a controlled release of an angiogenic peptide after cerebral ischemia-reperfusion injury (19), and growth-factor-loaded hydrogels demonstrate a coordinated microglial state, angiogenesis and neuroplasticity (20). Releasing a GSK3β inhibitor before VEGF further proves that the sequence can be intentionally structured instead of taken for granted (21). At later stages, micropore-forming microgel scaffolds can support neural progenitor transplantation and vascularization (22).

The lesson is that the duration of hydrogel presence should align with the biological phase. A quick response is beneficial when oxidative or inflammatory escalation is the main limiting factor, while extended presence is more advantageous for subacute niche conditioning and tissue reconstruction. Yet, excessive persistence might enhance foreign-body responses, disrupt tissue remodeling, or prolong the exposure to catalytic components after their optimal period.

### 3.3 Integration architectures and the central evidence gap

There are four key integration architectures. Physical encapsulation is straightforward and can minimize washout, but issues like nanoparticle leakage and diffusion barriers might separate substrate access from catalytic function. Covalent or affinity anchoring creates stable catalytic domains and may improve long-term local control, although immobilization can alter active-site accessibility. After delivery, *in situ* assembly allows injectable precursors to gel and adapt to the lesion. Multi-input or logic-gated integration uses pathological combinations to control exposure, degradation, or catalytic state.

An important demonstration of engineering feasibility is provided by a thermoresponsive *in situ* hydrogel integrated with ceria nanozyme, which enhanced the viability and paracrine function of mesenchymal stem cells in spinal cord repair (23). This study is not stroke evidence and should not be presented as such. Its significance is more limited but crucial: it demonstrates that catalytic nanoparticles can be incorporated into a soft, *in situ*-forming CNS matrix while maintaining a biologically relevant function. By contrast, the stroke literature cited above demonstrates advanced nanozymes and advanced responsive hydrogels largely in parallel. Thus, the most defensible conclusion is that the field possesses a robust mechanistic basis and multiple compatible components, yet there is limited direct validation of the entire integrated concept in stroke. This distinction is maintained throughout the review so that engineering feasibility is not conflated with stroke-specific therapeutic efficacy.

For future systems, logic gating should be implemented at a functional level rather than as decorative chemistry. For situations where either severe oxidative stress or acidity should activate an emergency release, an OR gate could be appropriate, whereas an AND gate might improve lesion selectivity by demanding both signals; a sequential gate may expose a catalyst first and a regenerative cue only after matrix cleavage. The important experiment is not to show the existence of two responsive bonds, but to demonstrate that the combined rule alters therapeutic timing *in vivo* and improves the benefit-risk profile over single-input controls.

This gap should shape experimental priorities. For a credible stroke nanozyme-hydrogel study, the integrated system should be compared to the nanozyme alone, a blank hydrogel, a hydrogel with a control nanoparticle that is non-catalytic, and a formulation that does not respond. In the absence of these controls, distinguishing synergy from basic additive retention is not feasible. The study should evaluate catalytic activity after gelation, particle release during degradation, and functionality in media similar to lesions.

## 4 Spatiotemporal control of the neurovascular unit

### 4.1 Hyperacute and acute phases: protect the reperfusion interface

The primary therapeutic obstacle is the reperfusion interface, not neurons on their own. The redox defense in endothelial cells is directly connected to the integrity of the blood-brain barrier and long-term recovery, as demonstrated by the need for endothelial peroxiredoxin-4 after ischemia stroke (24). Barrier failure is also linked to transcytosis, where glycocalyx loss can boost caveolin-1-dependent endothelial transport, independent of the straightforward “tight-junction opening” model (25). Assessment of a phase-matched system should include microvascular perfusion, permeability, edema, and inflammatory trafficking, as well as neuronal ROS markers.

For early use, the material must activate rapidly and fit the clinical workflow. This supports the use of small catalytic systems that can be delivered systemically or intra-arterially as supplements to reperfusion, as a large intracerebral hydrogel is seldom suitable for the initial minutes or hours of emergency treatment. Local hydrogels could still be applicable following thrombectomy or in certain delayed procedures, but the method of administration needs to be justified rather than considered just a formulation detail. An optimal early integrated system would deliver high catalytic activity during the oxidative burst and then decrease as the lesion begins to heal.

### 4.2 Subacute and chronic phases: recalibrate glia and enable reconstruction

BBB repair involves various cell types. Astrocytes provide metabolic support to endothelial cells, which can impact barrier integrity, including via mitochondrial transfer (26). Microglia and astrocytes are also part of a developing communication network after stroke (27). Both persistent astrogliosis and glial scar formation can contain injury and limit regeneration (28). As a result, aiming for “anti-inflammatory” effects is an overly simplistic goal. Continuous broad suppression might hinder debris removal, support for growth, and tissue remodeling.

In this later timeframe, hydrogel-based local therapy is more effective as the residence and matrix structure serve as benefits. Exosome-loaded adhesive hydrogels can facilitate cerebral angiogenesis and neurological improvement (29), and porous scaffolds can support cell infiltration and vascularization (22). A prospective integrated system could start with early catalytic buffering to stop redox-driven inflammatory amplification, then move to a lower catalytic intensity while revealing pro-angiogenic or matrix-supportive cues. In practice, NVU reprogramming signifies not the concurrent stimulation of every advantageous pathway, but an organized transformation in the prevailing material function.

## 5 Discussion and outlook

### 5.1 Match phase, route, and material function

The primary mistake in this area is considering multifunctionality as synonymous with therapeutic intelligence. A formulation with several active components doesn't automatically mean it's phase-specific. In acute stroke care, the emphasis is on rapid imaging, thrombolysis for patients who meet the criteria, and endovascular therapy for selected large-vessel occlusions, so supplementary materials must align with these logistics (1). CNS delivery remains a major translational constraint (30). For immediate use, nanozyme formulations that are systemic or compatible with endovascular methods are more feasible than implanting hydrogels directly into the brain. For conditioning cavities in the subacute phase, delivering cells, or long-term tissue remodeling, injectable local hydrogels are more suitable. The strongest development programs may therefore be modular rather than forcing every function into one formulation.

### 5.2 Define catalytic precision, degradation, and manufacturability together

It is essential to evaluate catalytic activity and material fate together. Trackable hydrogels, like those with MRI-detectable hyaluronic acid systems following intracerebral injection, exemplify how distribution and degradation can be directly monitored instead of inferred (31). In a broader sense, studies on biomaterial degradation still vary greatly in methods and chemical resolution (32). For nanozyme-hydrogel systems a minimum characterization package should quantify: the catalytic kinetics prior to and following gelation; activity under pH and protein conditions relevant to lesions; nanoparticle release and speciation as degradation occurs; residual metals or catalytic products in the brain and other organs; and functional decline.

The design of assays requires careful examination. Colorimetric enzyme-mimetic assays conducted in basic buffers might exaggerate their relevance in living organisms, and certain nanozymes may alter their perceived catalytic direction based on pH or substrate presence. Therefore, studies on stroke should correlate physicochemical kinetics with mechanism-related biomarkers in tissues and explicitly assess whether the material sustains the physiological redox signaling required for angiogenesis and immune resolution. “More ROS scavenging” is not, by itself, a translational endpoint.

There is a translational cost associated with complexity. Biomaterials engineered for clinical trials frequently face ongoing obstacles related to manufacturing consistency, sterilization, storage, scaling up, and regulatory classification (33). Future designs should focus on the most straightforward architecture that offers a clear benefit compared to using just nanozyme or hydrogel. Understanding how structure, properties, and function relate is more convincing than merely listing numerous components.

### 5.3 Raise the preclinical evidence standard

Stroke translation has repeatedly failed when efficacy is derived from narrow, single-laboratory models. STAIR XI advocated for a fundamental change in cerebroprotection research (34), whereas the Stroke Preclinical Assessment Network set up multicenter, randomized, and blinded frameworks for evaluating candidates (35). A subsequent multilaboratory trial demonstrated the feasibility of this approach (36), and the PRISM collective has extended the case for harmonized multicenter preclinical testing (37). Studies on stroke using biomaterials demonstrate positive cumulative effects; however, a systematic review has pointed out methodological limitations and publication bias (38).

Consequently, an integrated nanozyme-hydrogel platform should not progress based only on the infarct volume observed in young male rodents. Efficacy should be investigated at minimum across biological sex, age or comorbidity where relevant, reperfusion and non-reperfusion contexts, and clinically meaningful treatment delays. Endpoints should include behavior, imaging, BBB function, vascular remodeling, material fate and chronic safety. The principles of particle-based delivery used in stroke nanomedicine can guide this framework (39), and the progress in nanozyme biocatalysis highlights the importance of thorough structure-activity analysis instead of depending on a simple enzyme-like labels (40).

### 5.4 Outlook

Nanozyme-hydrogel systems that respond to stimuli provide a unified solution to a genuine issue: ischemic stroke is dynamic, while most treatments remain temporally static. Nanozymes can provide catalytic persistence and tunable redox control; by providing localized delivery, cue-responsive release, and a repair matrix, hydrogels could potentially bridge early reperfusion protection with later NVU restoration.

The field should, however, resist making premature claims. Direct evidence specific to strokes for fully integrated systems is still limited, and demonstrations across different indications cannot replace stroke validation. The next decisive studies are therefore not those with the greatest number of functions, but those that prove three points: combining the modules offers advantages that surpass the benefits of each one individually; the catalytic output adjusts according to the lesion phase; and the method, degradation characteristics, and production process align with a feasible clinical pathway. Meeting these criteria would move the concept from an attractive materials narrative toward a testable strategy for precision stroke therapy. The practical value of this framework is therefore its testable design logic, not an assumption that integration alone guarantees benefit.
